# A Comprehensive Review of Vaccine Development: From Traditional Platforms to Messenger RNA (mRNA) Technologies

**DOI:** 10.7759/cureus.100608

**Published:** 2026-01-02

**Authors:** Bhupendra Pawar, Subha Loganathan, Karthik Mukkatira Belliappa, Leela Bhavani Ranganathan, Komal Pukur Thekdi, Sanket Dadarao Hiware

**Affiliations:** 1 Department of Pharmacology, Vardhman Mahavir Medical College and Safdarjung Hospital, New Delhi, IND; 2 Department of Plant Breeding and Genetics, Agricultural Research Station, Tamil Nadu Agricultural University (TNAU), Pattukkottai, IND; 3 Department of Microbiology, Kurunji Venkatramana Gowda (KVG) Medical College and Hospital, Sullia, IND; 4 Department of Physiology, Sree Balaji Medical College and Hospital, Chennai, IND; 5 Department of Community Medicine, Dr. N.D. Desai Faculty of Medical Science and Research, Dharmsinh Desai University, Nadiad, IND; 6 Department of Anatomy, Graphic Era Institute of Medical Sciences, Dehradun, IND

**Keywords:** adaptive immunity, comparative vaccine efficacy, mrna vaccine platforms, next-generation immunization, vaccine equity and access

## Abstract

The trend of vaccine development over the last few years is that the traditional platform has been abandoned and moved towards newer modalities, and the current COVID-19 pandemic has accelerated this shift. Classical approaches (such as inactivated, live attenuated, and recombinant) can be shown to have reduced the burden of infectious diseases; however, they are also limited by their inability to scale production and prolonged development, as well as cold-chain limitations. The lacks became apparent through the pandemic and drove the demand for more agile, adaptive technology that COVID-19 has highlighted. The current review questions the goal of streamlining the immunization approaches in the face of emerging pathogens through a structured narrative comparison of immunogenicity, safety, efficacy, and platform stability of modern vaccination platforms within a narrative review framework. This analysis is anchored by a narrative synthesis of immunological, regulatory, and clinical literature between 2018 and 2025. Messenger RNA (mRNA)-based vaccines have demonstrated strong immunogenicity and practical efficacy in the real world, as well as varying safety profiles across platforms and the potential of new modalities that will be developed both to treat infectious diseases and to be applied in other contexts. The discussion also dwells upon the flexibility of regulation, unequal distribution, and surveillance-based pharmacovigilance. This review can be used to compile a thoroughly comparative view of modern vaccines, based on bringing together mechanistic insights and implementation barriers, which can guide future innovation and policy reconciliation and global resilience. It makes inferences in support of the more progressive, equity-based paradigm of pandemic preparedness and immunization strategy in the 21st century. This review highlights how mRNA technology has transformed the landscape of vaccinology, offering a foundation for faster, safer, and more equitable global immunization strategies in the post-pandemic era.

## Introduction and background

Immunization has been one of the most revolutionary interventions in the history of medicine, since it has had an enormous effect in reducing the burden of infectious diseases worldwide [1]. Ever since Edward Jenner successfully applied the smallpox vaccine in 1796, vaccinology has undergone several technological paradigms [2]. Live attenuated, inactivated, and toxoid-based vaccines make up the bulk of classical platforms of immunization programs globally [3]. These strategies are used because they resemble natural infection, generating protective and durable immunity. They have contributed to the elimination of smallpox, near-eradication of polio, and effective control of measles, diphtheria, and pertussis [4]. However, despite their success, these vaccines have limitations. They are usually associated with long development timelines, complex biological production processes, and sensitive cold-chain logistics [5]. Moreover, safety concerns, such as reversion to virulence in live attenuated vaccines and poor immunogenicity in inactivated formulation, also persist [6].

The global health impact of vaccines remains profound. According to the World Health Organization, immunization prevents 3.5-5 million deaths annually, yet in 2024, about 14.3 million unvaccinated children missed all vaccines. Global coverage reached 85% for DTP3, 84% for measles first dose, 31% for human papillomavirus (HPV) first dose in girls, and 52% for yellow fever in at-risk countries, underscoring persistent inequities in access [7]. Nevertheless, over the past two decades, an upsurge in re-emerging and evolving pathogens, including Zika virus, Ebola virus, and SARS-CoV-2, exposed critical weaknesses in the existing vaccine development landscape [8]. The unprecedented urgency of the COVID-19 pandemic triggered a paradigm shift toward faster, scalable, and adaptive technologies. This demand for agility accelerated innovation in vaccinology, enabling next-generation platforms to move from research into clinical practice at record speed [9]. Among these, messenger RNA (mRNA) vaccines emerged as a breakthrough. Although decades of research underpinned mRNA-based therapeutics, their clinical deployment during the pandemic was enabled by advances in RNA stabilization, lipid nanoparticle (LNP) delivery systems, and large-scale manufacturing [10]. Vaccines with more than 90% efficacy, including BNT162b2 (Pfizer-BioNTech; 95% CI 90.3-97.6%) and mRNA-1273 (Moderna; 94.1% CI 89.3-96.8%), were developed, approved, and distributed in months, an achievement unprecedented in the history of vaccinology [10]. By contrast, viral vector vaccines demonstrated efficacy ranges between 60% and 80%, and inactivated vaccine platforms typically achieved 50-70% efficacy, consistent with published phase 3 trial data [8,11].

mRNA vaccines are superior to conventional platforms in several ways. They offer cell-free, fully synthetic production, rapid adaptability of antigen design, low biosafety risk, and high immunogenic precision [10]. Recent developments in computational vaccinology and artificial intelligence (AI) have further advanced vaccine discovery pipelines. Machine learning-based frameworks and structural modeling approaches now facilitate the rapid identification of antigenic regions, improving vaccine target design and immune-response prediction [12]. These AI-assisted methods are increasingly integrated into mRNA vaccine research, enabling precision antigen engineering and accelerating preclinical validation. Beyond conventional linear mRNA, emerging RNA platforms such as self-amplifying RNA (saRNA) and circular RNA (circRNA) are being explored for enhanced stability, lower dose requirements, and prolonged antigen expression. Promising preclinical and clinical findings highlight their potential in both infectious disease and oncologic applications [13]. Mechanically, mRNA vaccines elicit both cell-mediated and humoral immunity, and most induce a desirable Th1-biased response.

**Figure 1:**
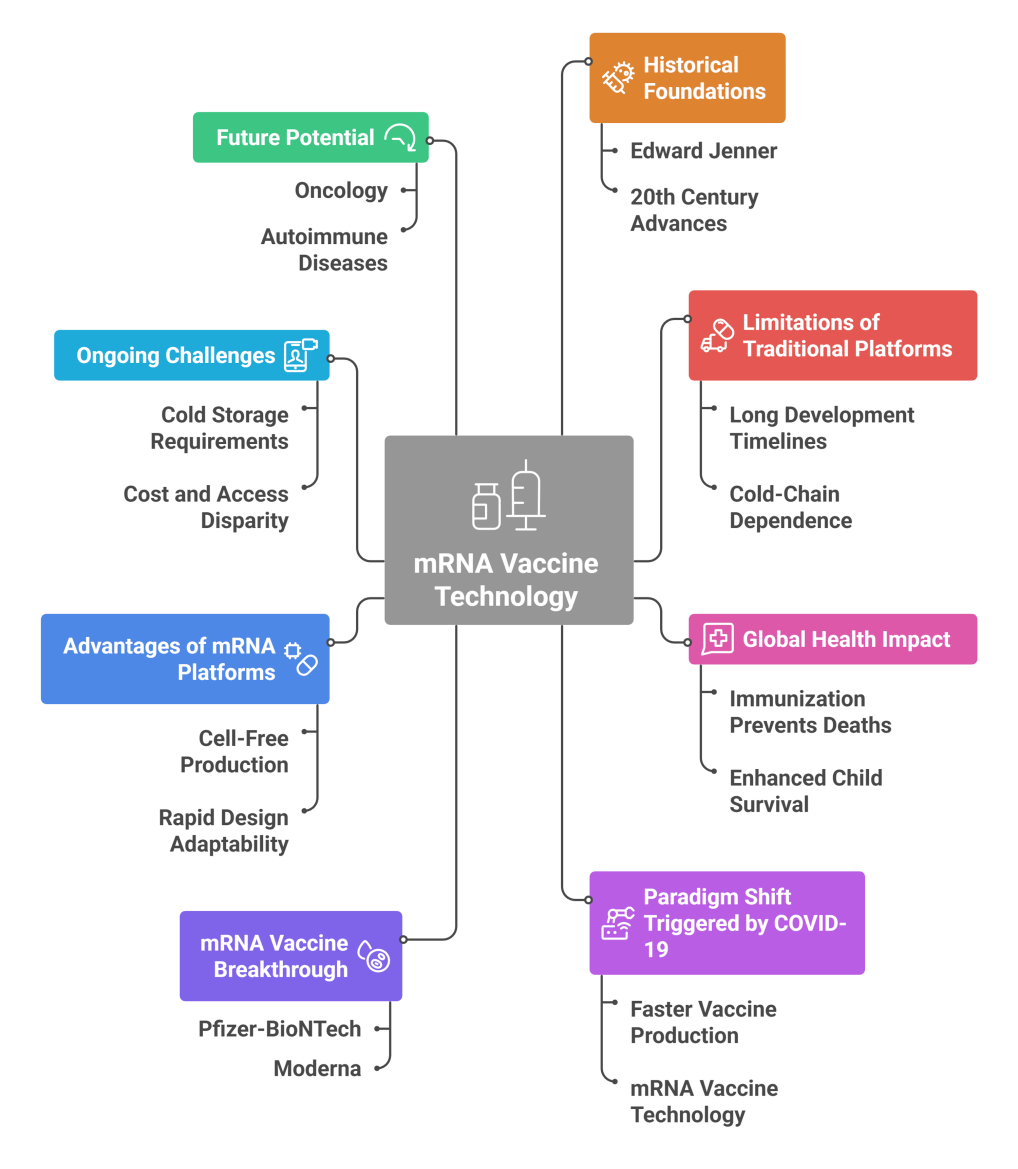
Mapping the evolution and impact of mRNA vaccine development Image Credit: Authors

However, challenges persist. The need for ultra-cold storage, variable expression profiles, rare immune-mediated adverse events, and disparities in manufacturing capacity between high- and low-income nations remain critical concerns [9]. Moreover, the durability of protection and optimization of booster strategies require continued long-term study. In addition to infectious diseases, mRNA vaccination platforms are now being investigated for oncological therapies, autoimmune modulation, and personalized immunotherapeutics. Their ability to be multiplexed, rapidly prototyped, and mass-produced fundamentally redefines how humanity can respond to future biological threats [8]. Figure 1 summarizes the transition from traditional to mRNA vaccine platforms, highlighting key milestones, advantages, challenges, and future potential shaping modern immunization strategies.

Objectives of the review

The present review critically analyzes the process of vaccine development, tracing its evolution from traditional platforms to the emergence of mRNA-based technologies. It compiles up-to-date evidence to elucidate the immunological rationale, safety data, efficacy, and scalability of diverse vaccine modalities. Particular emphasis is placed on how the COVID-19 pandemic accelerated the rapid development and global deployment of mRNA vaccines, assessing their implications for the future of vaccinology. The review also addresses regulatory and methodological aspects, including study design frameworks, clinical endpoints, and ethical considerations within expedited development settings. Furthermore, it examines the limitations of existing vaccine technologies and offers evidence-based recommendations to enhance equity, innovation, and global preparedness. Through this synthesis, the review aims to guide researchers, clinicians, public health professionals, and policymakers in understanding the evolving landscape of vaccine science and its influence on global health in the 21st century.

Scope and methodology

To provide a clear orientation for readers, this review first examines classical vaccine types, including live attenuated, inactivated, and toxoid-based platforms, followed by recombinant, viral vector, and nucleic acid (DNA and mRNA) technologies. It then discusses comparative immunogenicity, safety profiles, and regulatory aspects, concluding with future perspectives and ethical considerations. 

This work is a narrative literature review, not a systematic review or meta-analysis. It aims to critically synthesize published evidence on traditional and next-generation vaccine platforms without performing quantitative pooling or formal statistical comparisons. The literature search was performed in PubMed, Scopus, and Web of Science, covering studies published between January 2018 and April 2025. Boolean search strings combined controlled vocabulary and free-text terms such as ("vaccine development" OR "vaccine platform" OR "mRNA vaccine" OR "DNA vaccine" OR "viral vector vaccine" OR "computational vaccinology" OR "AI-assisted vaccine design"). The most recent search was conducted in August 2025.

Peer-reviewed journal articles, review papers, and authoritative institutional reports focusing on vaccine mechanisms, clinical efficacy, safety, and translational applications were included. Preprints, conference abstracts, and non-peer-reviewed sources were excluded to ensure scientific rigor. Titles and abstracts were screened to ensure relevance, and full texts were reviewed for inclusion. Key findings were synthesized narratively under thematic sections, such as technological evolution, comparative immunogenicity, and implementation challenges. Because this is a narrative review, no formal risk-of-bias scoring was applied; however, only peer-reviewed and reputable sources were included to minimize interpretive bias.

Statistical considerations

This review did not perform any quantitative synthesis, meta-analysis, or statistical pooling. Reported efficacy or safety percentages are cited directly from the respective clinical trial publications or authoritative regulatory data. Confidence intervals (CIs) and statistical metrics are included only where explicitly reported in the original studies to preserve the accuracy of comparative data and maintain scientific integrity.

## Review

Classical vaccine platforms

Classical vaccines are the pillars of contemporary immunoprophylaxis. These include inactivated (killed), live attenuated, and toxoid-based vaccines. The usage of these platforms presents their strategies to present antigens to the host immune system and resemble an infection to induce immunological memory [11]. Inactivated vaccines make use of the whole pathogen that has been made non-replicative either by the use of chemical substances like formaldehyde or beta propionate lactone or by physical means like heat and radiation [12]. Although these are not replicated, they have enough antigenic integrity to provoke immune recognition. Some of them are inactivated poliovirus vaccine (IPV) and hepatitis A vaccines [13]. These preparations usually need repeated doses and adjuvants in order to bring out strong immunity.

In comparison, weak, replicable strains of the pathogen are used in live attenuated vaccinations, in the host, and have no complete virulence. Serial passage of a non-human cell line or genetic modification of attenuation is used [14]. Two well-known examples are the measles, mumps, and rubella (MMR) combination and the oral polio vaccine (OPV). These vaccines induce long-lasting immune responses at mucosal, humoral, and cellular levels. Nevertheless, precautions should be taken in their application to immunocompromised persons as there is a documented albeit rare case of reversion to virulence, including vaccine-associated paralytic poliomyelitis (VAPP) in the case of OPV vaccinees [15]. Toxoid vaccines go against the bacteria's toxins and not the bacteria. These vaccines have killed exotoxins, which preserve immunogenicity and get rid of toxicity. The diphtheria and tetanus parts of the diphtheria-tetanus-pertussis (DTP) vaccine are a good example [14].

The major strengths of classical platforms are that they have a long history of safety, are relatively inexpensive, and have well-studied manufacturing procedures. Indirect protection also occurs in some instances where they use live attenuated vaccines, as was the case with OPV, and which contributes to the herd immunity by secondary spreading the attenuated strain [14]. Nonetheless, such platforms have some shortcomings, including cold-chain and the necessity of booster doses because of the declining immunity. Moreover, inactivated and toxoid vaccines usually need adjuvants and fail to induce robust cellular immune responses, and so they have to be improved for particular groups of individuals [12].

Recombinant and subunit vaccines

The recombinant vaccines and subunit vaccines signal a revolution in antigen design. In contrast with whole-pathogen vaccines, these platforms use particular antigenic parts that tend to be peptides or proteins, which are adequate to provoke reactions from the immune system without adding infectious material [16]. Subunit vaccines are vaccines that contain purified antigenic structures of the pathogen, e.g., surface proteins or viral envelope proteins. They can be produced with the help of recombinant DNA technology with the assistance of yeast, insect, or mammalian cell expression systems [16]. The first licensed recombinant vaccine was the hepatitis B surface antigen (HBsAg) vaccine that was made in *Saccharomyces cerevisiae*. On the same note, vaccines against human papillomavirus (HPV) use virus-like particles (VLPs) made of protein L1, which in turn self-assemble to form highly immunogenic, non-infectious capsids [17].

These platforms are noted for their favorable safety profiles. They are applicable in immunocompromised people because they lack live organisms or replicative factors [18]. Also, the recombinant approaches permit extremely accurate control of both antigen structure and antigen content, permitting batch-to-batch consistency. Subunit vaccines, however, tend to be less immunogenic than live or whole-cell formulations because they cannot replicate or stimulate innate immune pathways in a strong manner [18]. Consequently, they usually need adjuvants, immunostimulants which intensify the host reaction. The more common adjuvants are aluminum salts (alum), AS04 (alum plus monophosphoryl lipid A), and MF59 (an oil-in-water emulsion) [19].

**Figure 2:**
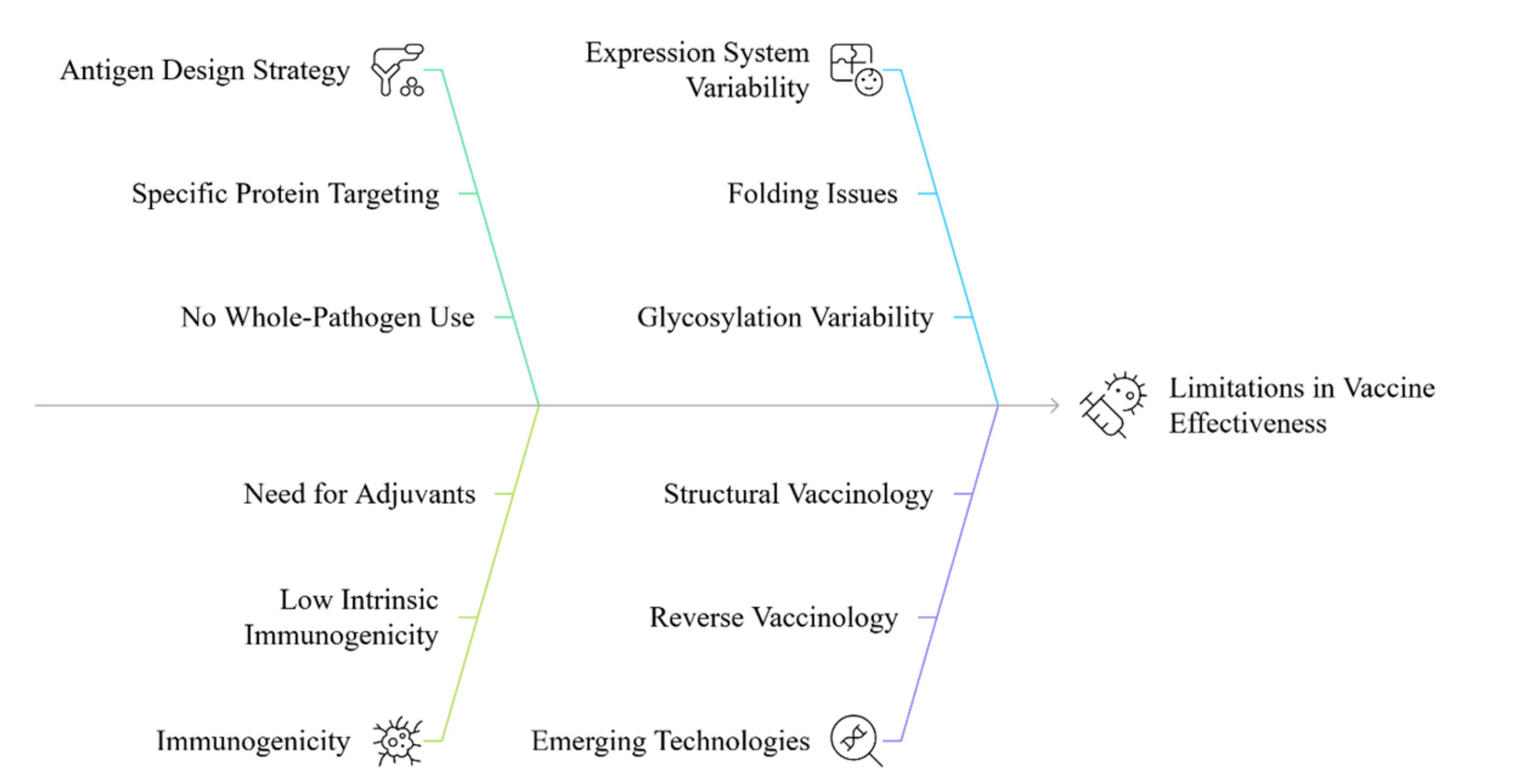
Determinants contributing to limitations in vaccine effectiveness Image Credit: Authors

The other disadvantage is that it often needs booster immunization to maintain the protective immunity over the period [20]. In addition, expression of antigen as well as folding, glycosylation, and presentation of epitopes may be different in various expression systems, and this could impact the vaccine's immunogenicity and effectiveness [16]. New developments in this area are reverse vaccinology and structural vaccinology, where genomic and proteomic technologies are used to define conserved antigenic determinants to enable rational vaccine design [19]. These methods are being directed to such complex pathogens as HIV and malaria, where antigenic variety poses a significant challenge. Subunit and recombinant vaccines are extremely safe and can be highly molecularly precise. They, however, frequently need adjuvants and complex delivery plans to induce long-term immune responses, especially in immunocompromised populations or with antigens of low intrinsic immunogenicity. Figure 2 depicts the multifactorial determinants that impact the performance of recombinant and subunit vaccines, highlighting antigen design strategy, immunogenic limitations, expression system variability, and emerging technological innovations such as structural and reverse vaccinology.

Viral vector-based vaccines

Viral Vectors

Immunization using viral vectors is based on modified viruses as carriers of genetic information coding antigenic proteins. These vectors assist in the intracellular expression of the target antigen, and this allows both humoral and cellular immune responses [21]. Viral vectors are divided into two primary categories, namely, non-reproducing and replicating viral vectors. Replicating vectors, i.e., vesicular stomatitis virus (VSV), replicate in host cells, increasing antigen load and immunostimulation. The vectors (e.g., human or simian adenoviruses, e.g., ChAdOx1 in AstraZeneca COVID-19 vaccine) that do not replicate are modified to eliminate critical genes needed to replicate them and enhance their safety [21]. Following vector entrance into the host cell, the encoded antigen is produced endogenously and presented via the major histocompatibility complex (MHC) class I and II pathways, which subsequently trigger cytotoxic T-cell responses and antibody formation [22]. It is an aspect that makes viral vector vaccines especially useful in situations that demand high cellular immunity.

The use of adenovirus-based vaccines (Ad26.COV2.S, Johnson & Johnson; ChAdOx1 nCoV-19, AstraZeneca; Gam-COVID-Vac, Sputnik V) in response to the COVID-19 pandemic proved that this vaccine type could be used to massively immunize based on a relatively short development period [21]. Viral vector vaccines offer several logistical and immunological advantages. They usually need fewer doses as they are highly immunogenic and their relative thermostability (usually within 28°C) allows their distribution in resource-poor contexts [23]. Besides, the vector by itself can act as an intrinsic adjuvant, boosting the immune activation via pattern recognition receptors [22].

However, several challenges persist. A major constraint is pre-existing immunity against the vector, particularly to popular adenovirus serotypes such as Ad5 that may neutralize the vector before antigen administration [24]. The combination of the rare human serotypes and the use of Simian adenoviruses or heterologous prime-boost (involves the use of different vectors in successive doses) strategies are some of the ways of overcoming this [24]. Moreover, there are possible outcomes of host immune reactions raised against the vector during the initial dose that can inhibit the success of other doses, forming an immunogenic ceiling [24]. The size of the vectors limits the number or complexity of antigens that may be encoded, and this may limit applications that need multivalent coverage. Regulatory aspects are also involved since most viral vectors are genetically modified organisms (GMOs). Regulatory aspects include the stringent requirements of GMOs, many of which are viral vectors. This adds more safety checks and questioning of the masses, especially in areas where there is low acceptance of the genetic technologies [25].

DNA vaccines

Mechanism and Clinical Progress

DNA vaccines represent a next-generation approach within nucleic acid-based immunization platforms. The mechanism of action of these vaccines is the introduction of circular plasmid DNA encoding a target antigen into the cells of the host and its transcription into mRNA and translation into antigen proteins, which are finally presented through MHC class I and II pathways as a way of inducing adaptive immune responses [26]. Designed to mimic the immunological hallmarks of natural infection, DNA vaccines aim to stimulate both humoral and cellular immunity [27]. While preclinical studies in animal models have shown robust immunogenicity, the translation to consistent efficacy in humans has been limited, primarily due to challenges in nuclear translocation and low transfection efficiency [28].

Clinically, DNA vaccines are currently being evaluated in humans for diseases like Zika, HIV, and COVID-19, with some candidates progressing to phase 2 and 3 trials [8,29]. They have also been approved for use in veterinary settings, such as for West Nile virus in horses and infectious hematopoietic necrosis virus in fish [29]. Table 1 summarizes the platform's core mechanisms, delivery methods, immunogenic outcomes, current applications, and development status. Despite their limitations, DNA vaccines remain attractive due to their safety, rapid design potential, and manufacturing scalability, particularly for future outbreaks and prime-boost strategies.

**Table 1:** Overview of DNA vaccines: mechanism, application, and development status MHC: major histocompatibility complex

Parameter	Description	Reference
Vaccine platform	Plasmid DNA-based nucleic acid vaccine	[26]
Mechanism of action	In order to trigger humoral and cellular reactions, plasmid DNA is injected intramuscularly or intradermally into host cells, enters the nucleus, is converted to mRNA, is translated into antigenic protein, and then is presented via the MHC I and II pathways	[7]
Immune response type	Mimics a natural infection by inducing humoral antibody production and cellular T-cell activation	[20]
Delivery method	Primarily intramuscular or intradermal injection	[16]
Preclinical efficacy	Demonstrates robust immunogenicity in animal models	[24]
Human clinical progress	Modest efficacy in humans; ongoing trials for Zika, HIV, and COVID-19, including phase 2 and 3 candidates	[9]
Approved veterinary use	Licensed for veterinary diseases, including West Nile virus (horses) and infectious hematopoietic necrosis virus (fish)	[18]
Advantages	Safe, non-replicating; induces both arms of adaptive immunity; scalable and cost-effective production; easy to customize	[2]
Limitations	Poor transfection efficiency in humans, suboptimal immunogenicity, and limited clinical translation require improved delivery systems	[21]

Advantages and Technical Challenges

Some of the outstanding benefits of DNA vaccines are that they are thermally stable and effectively do not require ultra-low-temperature storage and distribution in resource-poor environments [30]. Their manufacturing is cell-free, scalable, and not expensive and depends on fermentation in bacteria and purification of plasmids, instead of pathogen growth and protein expression systems [31]. DNA vaccines, however, are foiled by poor transfection levels in human cells, particularly when administered through the conventional needle injection. This limits intracellular antigen production and overall immunogenicity [32]. To increase delivery, techniques like electroporation, gene guns, and microneedle arrays have been developed, but scalability and patient tolerance are still an issue [33]. The other issue is associated with the potential of genomic integration, but there is no genotoxicity reported in human studies so far [34]. There is also a chance that immunogenicity may not be up to the mark because of the inefficient expression of the antigen or the degradation of the plasmid. Strategy of vector design, such as codon optimization and powerful eukaryotic promoters, is used to enhance the kinetics of expression and immune activation [35]. DNA vaccines are also an interesting platform with a good prospect of pandemic response and chronic infections, given the ongoing rapid advancements in delivery technologies and vector engineering [36].

mRNA vaccine technology: foundations

Platform Architecture and Delivery

The mRNA vaccines have transformed the field of vaccinology due to their modularity, safety, and rapidity. They present synthetic mRNA of a target antigen to the host cells in which the antigen is expressed and identified by the immune system [26]. This intracellular expression of an antigen is a simulation of viral infection and therefore acts as a priming of an adaptive immune response. The idea of mRNA-based immunization was first introduced at the beginning of the 1990s; however, clinical translation faced limitations due to the instability, poor delivery, and undesirable activation of the innate immune system. These obstacles were surmounted with recent developments in molecular engineering and delivery vehicles, and the result is the successful roll-out of COVID-19 vaccines, including BNT162b2 (Pfizer-BioNTech) and mRNA-1273 (Moderna), which showed >90% and >94% efficacy in phase 3 trials, respectively [10]. There are two broad classes of mRNA vaccines in development, conventional (non-replicating) and self-amplifying mRNA (saRNA). Traditional mRNA just has the coding part of the antigen, whereas saRNA has viral replicase parts that permit RNA amplification inside the cell, and it can be dosed lower [27]. LNPs are the most effective mRNA delivery technique. Ionizable lipids, cholesterol, phospholipids, and PEG-lipids make up LNPs and act to shield the mRNA against degrading enzymes and also enable escape from the endosome following cell entry [28]. The endosomal membranes are caused by ionizable lipids at an acidic pH to lose their integrity through the acquisition of a positive charge and release into the cytosol [29].

Immunogenic Profile and Challenges

Among the most significant qualities of mRNA vaccines is a cell-free and in vivo fully synthetic production. This allows quick production, avoids cell culture or live viruses, and quickens response to new diseases [30]. mRNA vaccines generate both branches of adaptive immunity and stimulate neutralizing antibody synthesis and responses of cytotoxic T cells. They are non-integrating and biodegradable and, thus, have an excellent safety profile, suitable to be use in wide populations [31]. However, the problem still exists. mRNA is unstable and needs to be stored at low temperatures of 20°C or less. In addition, innate receptors, including TLR7 and RIG-I, are able to sense the immune system, thereby inducing inflammation and hindering the efficacy of translation [32]. Attempts to control such effects include chemically modified nucleosides and sequence engineering [33]. In general, mRNA vaccines are a potent, safe, speedy, and versatile bundle. The fact that they were successfully used as a clinical tool in the situation of the COVID-19 pandemic helps to mark their transformative potential in the background of viral and non-infectious illnesses [26].

Molecular engineering and optimization in mRNA vaccines

Structural Design Elements

The efficiency of mRNA vaccines is highly connected with the superior molecular engineering approaches that increase their stability, translation, and immunogenicity. Some of the main fields of optimization are codon usage, untranslated regions (UTRs), cap structures, poly(A) tails, and delivery systems [26]. Codon optimization is a process of choosing codons of great translation efficiency in human cells in order to maximize the production of proteins without changing the sequence of the amino acids [34]. This reduces the pause of ribosomes and enhances the general antigen synthesis rate.

The 5 UTR and the 3 UTR of the coding sequence are important in mRNA stability and translation. UTRs of naturally stable transcripts, e.g., human 6-globin or 6-globin genes, are normally used to prolong mRNA half-life and exonucleolytic digestion [35]. Ribosomal recruitment and mRNA stability rely on the 5′ cap structure. Anti-reverse cap analogs (ARCA) and CleanCap are modified cap analogs that are intended to enhance translation initiation with a reduced immune recognition [30]. Likewise, the optimized poly(A) tail increases mRNA nuclear export, translation, and stability, and optimal lengths are well-tuned in the design of vaccines [33]. Table 2 summarizes key molecular engineering and delivery strategies that enhance mRNA vaccines' immunogenicity, stability, and translational effectiveness, forming the foundation for their clinical success and versatility.

**Table 2:** Molecular optimization and delivery enhancements in mRNA vaccine design UTR: untranslated region; ARCA: anti-reverse cap analog; LNP: lipid nanoparticle; PRRs: pattern recognition receptors

Optimization domain	Strategy or feature	Functional benefit	Reference
Codon optimization	Preferential use of codons with high translation efficiency	Enhances protein yield without altering amino acid sequence	[34]
5′ and 3′ UTR engineering	Use of stable UTRs (e.g., human α-globin)	Prolongs mRNA half-life and improves ribosomal recruitment	[35]
Cap structure modification	ARCA, CleanCap	Promotes translation initiation and reduces innate immune activation	[30]
Poly(A) tail tuning	Optimal polyadenylation length	Increases nuclear export, stability, and translational efficiency	[33]
LNP delivery	Ionizable lipids enable endosomal escape via pH-sensitive membrane fusion	Facilitates cytosolic delivery while reducing degradation in the extracellular space	[28]
LNP composition refinement	Biocompatibility-improved lipids	Lowers inflammatory response and enhances tolerability	[26]
Stabilization techniques	Lyophilization; cryoprotectants (e.g., trehalose, sucrose)	Improves storage stability and shelf-life; reduces cold-chain dependency	[31]
Modified nucleosides	Pseudouridine, 5-methylcytidine	Evades PRRs and minimizes innate immune activation	[26]
Multivalent mRNA constructs	Encoding multiple antigens in a single transcript	Enables broad-spectrum or variant-inclusive vaccination (e.g., HIV, influenza)	[27]

Delivery, Stability, and Immune Tuning

The delivery of LNP systems is a modulator in addition to a carrier. LNPs contain ionizable lipids that are positively charged at an acidic endosomal pH, which enables membrane fusion and the release of mRNA into the cytosol [28]. Current developments are aimed at the modification of lipid composition towards biocompatibility and minimization of inflammatory effects [26]. Formulation science has also resulted in the improvement of storage and stability. To increase shelf-life and minimize the use of cold-chain, lyophilization and addition of cryoprotectants such as trehalose or sucrose are being applied [31].

Pseudouridine and 5-methylcytidine are added to synthetic mRNA to minimize the innate immune activation of such mRNA. These alterations overcome pattern recognition receptors and enhance translation [26]. Lastly, the modular vaccine design permits mRNA constructs to express several antigens in one shot, which permits multivalent vaccines against complex or quickly mutating pathogens, including HIV or influenza [27].

Comparative immunogenicity and efficacy

Immunogenicity is the ability of a vaccine to elicit a measurable immune response, commonly assessed by T-cell subset activation and neutralizing antibody production [37]. Strong, enduring immunity is produced by live attenuated vaccinations, such as those for yellow fever and measles, with high titers of virus-neutralizing antibodies and persistent cellular responses following a single dose [38]. By mimicking natural infection, they activate both humoral and cellular immune systems and typically provide lifelong protection. In contrast, inactivated vaccines generally produce lower magnitude immune responses, favoring humoral immunity with minimal cytotoxic T-cell activation [39]. Achieving protective levels often requires multiple doses and adjuvants, as seen with influenza and hepatitis A vaccines. Protein-based vaccines, like those for hepatitis B and HPV, elicit strong antibody titers, but their efficacy depends heavily on adjuvants such as AS04 and MF59 to enhance immunogenicity [40]. These vaccines tend to induce limited T-cell activity, potentially impacting long-term memory formation.

mRNA vaccines have demonstrated the ability to produce balanced, antigen-specific immune responses characterized by robust humoral and cellular immunity [10]. Delivered via LNPs, MHC class I and II pathways are used to present the antigenic proteins that are produced when the mRNA is translated within the host cells, inducing neutralizing antibodies and cytotoxic T-cell responses [26]. Notably, vaccines like BNT162b2 and mRNA-1273 are associated with polyfunctional T-cell responses, including IFN-γ secretion and cytotoxic activity, contributing to effective viral control [41]. These clinical outcomes align with consolidated evidence from comparative reviews in Nature Reviews Drug Discovery, which integrate efficacy and safety data across mRNA, DNA, and viral vector vaccine platforms [5,30]. Viral vector vaccines also enable intracellular antigen expression, enhancing antigen presentation and generating strong T-cell-mediated immunity [42]. However, previous exposure to the viral vector can reduce the immune response to booster doses due to anti-vector immunity [14]. DNA vaccines, though immunogenic in animals, often elicit weaker responses in humans because of inefficient nuclear translocation and suboptimal in vivo expression, necessitating delivery enhancements like electroporation [34].

Data from the COVID-19 pandemic has provided unprecedented comparative insight. mRNA vaccines consistently demonstrated over 90% efficacy against symptomatic disease in phase 3 trials and showed broad effectiveness across various demographic groups [10]. Adenoviral vector vaccines, such as ChAdOx1 and Ad26.COV2, showed intermediate efficacy of around 60-80%, while inactivated vaccines like CoronaVac and BBIBP-CorV reported efficacy in the range of 50-70% [43]. These findings are supported by real-world vaccination data from regions including Europe, North America, and Asia. Demographic factors significantly influence immune responses. Elderly individuals and those with immunosuppressive conditions tend to exhibit diminished responses, particularly to inactivated and subunit vaccines [13]. In such populations, heterologous prime-boost regimens, such as administering an adenoviral vector vaccine followed by an mRNA vaccine, have improved both antibody and T-cell responses [44]. These combinations leverage different immunological pathways and have been practical solutions in contexts with supply limitations.

**Figure 3:**
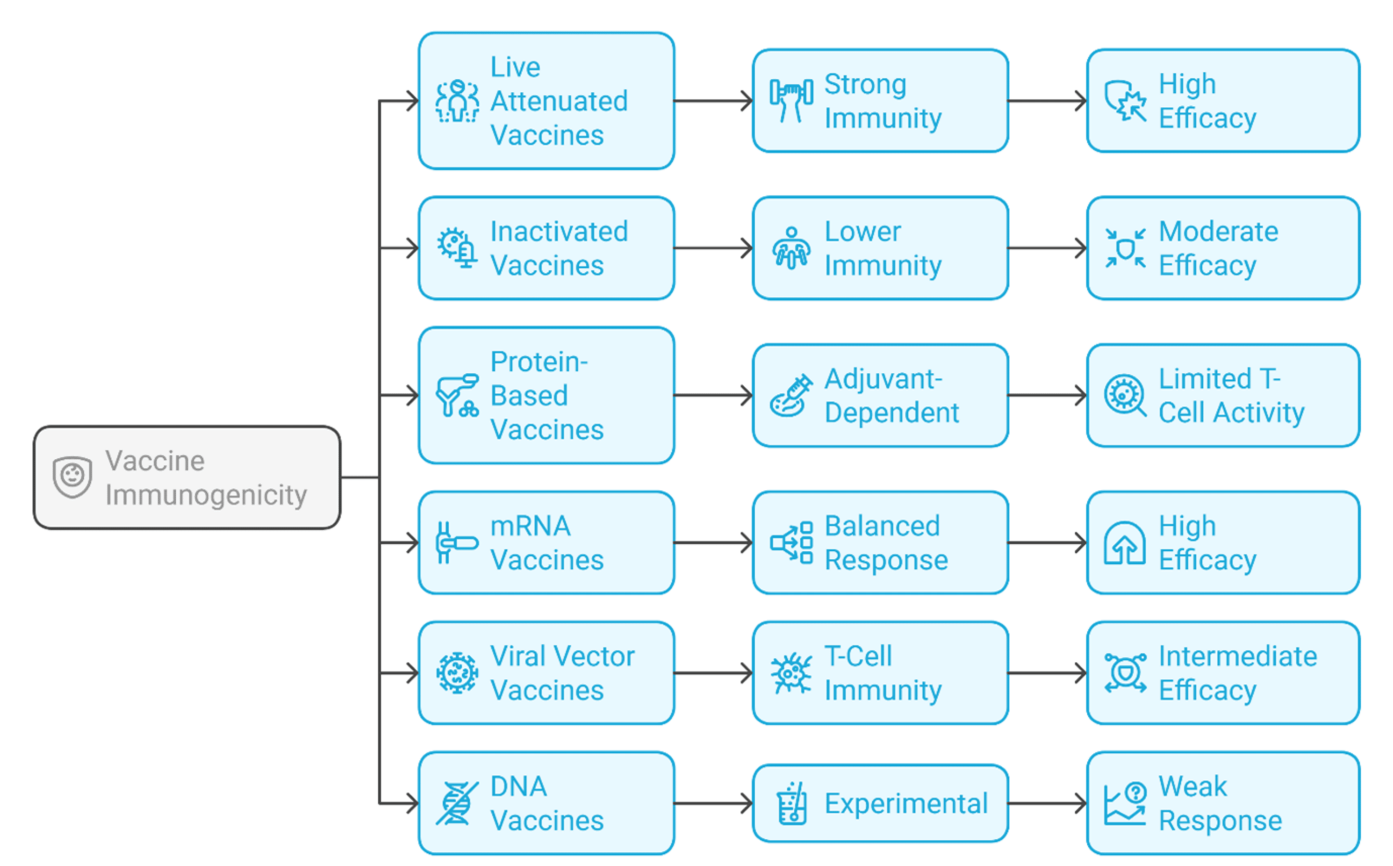
Comparative immunogenicity and efficacy profiles of vaccine platforms Image Credit: Authors

Durability of immunity is another critical factor. Antibody levels decline relatively quickly following inactivated, subunit, and mRNA vaccination, necessitating booster doses within months [20]. In contrast, live attenuated vaccines often confer durable protection without the need for additional doses [11]. mRNA vaccines, despite waning antibody titers, maintain T-cell responses that contribute to long-term protection against severe disease [44]. Viral vector vaccines also show declining antibody levels over time and face challenges with repeat dosing due to anti-vector immunity. DNA vaccines remain experimental in humans and have yet to show strong evidence of long-term protection in clinical trials [34]. Figure 3 illustrates the immunogenic strengths and efficacy outcomes of major vaccine types, highlighting variations in immune activation mechanisms and clinical performance across traditional and next-generation platforms.

Safety and adverse events

Safety is one of the core principles in vaccine development and deployment. Although all licensed vaccines undergo rigorous preclinical and clinical evaluation, platform-specific differences exist regarding the nature, frequency, and severity of adverse events [12]. Live attenuated vaccines, due to their replicative nature, are contraindicated in immunocompromised individuals and during pregnancy owing to the theoretical risk of reversion to virulence, as historically observed with oral polio VAPP [15]. Inactivated vaccines have a well-established safety profile, with most adverse events being mild and localized, such as injection site pain, redness, or transient fever [21]. Serious adverse events are rare and often attributable to vaccine excipients rather than the inactivated pathogen itself. Protein subunit and recombinant vaccines are similarly recognized for their excellent safety, particularly because they lack any replicative material [17]. However, the inclusion of newer adjuvants can lead to increased local reactogenicity or systemic symptoms like malaise and fatigue, depending on the formulation and dose [18].

The use of viral vectors introduces a distinct safety signature. Short-term systemic adverse effects such as headache, fever, and chills are relatively common following the first dose and typically resolve without intervention [23]. Of greater concern is vaccine-induced immune thrombotic thrombocytopenia (VITT), a rare but serious clotting disorder predominantly reported in younger adults, particularly females, after the administration of ChAdOx1 or Ad26-based vaccines [45]. Though the incidence varies by age and region, it remains very low and is outweighed by the benefits of vaccination, especially in high-transmission settings. Reported adverse events include rare cases of VITT following adenoviral vector vaccines and myocarditis associated with mRNA-based vaccines, both of which have been closely monitored through post-marketing surveillance frameworks [24,25,31].

The mRNA vaccine platform has demonstrated an excellent safety profile across age groups. The most frequently reported adverse effects are transient and include injection-site pain, myalgia, low-grade fever, and fatigue, typically resolving within 24-48 hours [10]. A rare adverse event linked to the LNP component is anaphylaxis, usually associated with polyethylene glycol (PEG), occurring between two and five cases per million doses, according to estimates, and managed effectively through standard emergency procedures [19]. Myocarditis has also emerged as a rare, mostly self-limiting complication, primarily affecting young males following the second dose [23]. DNA vaccines, though not yet widely implemented in human populations, have shown no significant adverse effects in clinical trials. Concerns about genomic integration remain theoretical, with no evidence of clinical genotoxicity to date. Nonetheless, long-term monitoring is essential to fully establish their genomic safety profile [34].

Post-marketing Surveillance and Autoimmunity

Autoimmune reactions have been a topic of interest in vaccinology due to mechanisms such as molecular mimicry and bystander activation. To date, no consistent evidence links vaccines with an increased incidence of systemic autoimmune diseases. However, isolated reports of Guillain-Barré syndrome, autoimmune thrombocytopenia, and lupus flare-ups following vaccination have been documented and warrant continued vigilance and research [20].

Post-marketing surveillance systems play a critical role in maintaining vaccine safety. In the United States, the Vaccine Adverse Event Reporting System (VAERS) and, in Europe, the EudraVigilance system collect and analyze adverse event data to detect early safety signals [24]. These platforms are supported by active surveillance tools, electronic health record integration, and advanced analytics to facilitate the real-time monitoring of rare events [25]. Furthermore, global pharmacovigilance efforts coordinated by the World Health Organization contribute to transparency and enable the prompt implementation of safety interventions when necessary.

Regulatory and ethical frameworks

Vaccines are developed, approved, and distributed through regulatory frameworks that are well-structured to provide safety, efficacy, and quality. However, the COVID-19 pandemic required major transformations of such processes to allow responding to such an unusual global health crisis in a timely manner [45]. Consequently, various regulatory agencies introduced fast-track procedures without scientific dilution. Mechanisms that were used by agencies like the US Food and Drug Administration (FDA) to accelerate data review and developer-regulator communication included Fast Track, Breakthrough Therapy, and Priority Review designations [46]. By implementing conditional marketing authorizations and rolling reviews, the European Medicines Agency (EMA) made it possible to quickly evaluate mRNA vaccines like mRNA-1273 and BNT162b2 [10].

The Emergency Use Authorization (EUA) frameworks were especially useful in enabling the temporary use of unapproved medical products to be used in cases where the perceived benefit was likely to supersede the known risk [19]. This instrument facilitated the deployment of the COVID-19 vaccines to the world in a few months after the publication of the phase 3 data. Nevertheless, the use of EUAs was also associated with the issues of informed consent, the lack of long-term safety data, and the differences in national regulatory standards. The pandemic also showed inequality in vaccine equity across the world. In spite of the early international initiative like the COVAX facility, the high-income countries obtained preferential access to vaccines through bilateral deals, and many low- and middle-income countries (LMICs) were confronted with late and inadequate vaccine deliveries [47]. Additional access disparities were perpetuated by structural obstacles like cold-chain constraints, regulatory capacity deficits, and patent monopolies.

The conversation surrounding the intellectual property (IP) and the pandemic brought to the fore the conflict between the need to reward innovation and grant widespread access to it. Submissions of temporary IP waivers under the Agreement on Trade-Related Aspects of Intellectual Property Rights (TRIPS) of the World Trade Organization were meant to facilitate an increase in manufacturing capacity in LMICs [48]. These proposals faced opposition from some countries and industry players, but these ideas brought up the bigger debates on open licensing, patent pools, and technology transfer partnerships. Other than the IP barriers, the lack of harmonized regulatory standards became a challenge to the global rollout efforts. Regulatory fragmentation added to the time and complexity of global deployment of vaccines, especially in cases of emergency [24]. In addition, there was an ethical issue that covered trial representation as the underrepresentation of subjects in LMICs in the most important studies undermined the safety and efficacy results in various demographics [49].

In the future, the important thing is to reinforce the manufacturing infrastructure at the regional level, to broaden the data-sharing platforms, and to coordinate the processes of emergency authorization. These reforms should be conducted through ethical principles, which are equitable access, a focus on high-risk populations, and openness in resource distribution. The global health response in the future should not view vaccines as commodities but as a global public good that is based on solidarity, equity, and justice [26].

Applications beyond infectious diseases

The effectiveness of mRNA vaccines against COVID-19 has triggered an evolutionary change to the field of vaccinology, proving the platform to be more versatile, adaptable, and scalable and therapeutically applicable to non-infectious conditions. Oncology is one of the most promising fields of development in which mRNA vaccines are under development to target the tumor-specific mutations. Vaccines against cancer are made to induce the immune system to target neoantigens, tumor-associated antigens (TAAs), or mutations specific to a patient's tumor, and as such, mRNA technology ensures a patient-specific vaccine can be designed rapidly in response to tumor sequencing [49]. As such, melanoma and non-small cell lung cancer (NSCLC) have individualized mRNA vaccine clinical trials, and both demonstrate a favorable safety and immunogenicity profile [30].

Together with immune checkpoint inhibitors, such as PD-1 or CTLA-4 antagonists, which increase anti-tumor immunity by removing the inhibitors of T-cell activation, these vaccines exert the greatest potential [50]. Such a synergistic strategy is especially promising in the circumvention of immune evasion mechanisms developed by aggressive tumors. Besides oncology, mRNA is used to create universal influenza vaccines as well. In contrast to the traditional flu vaccines, which have to be redesigned each year in accordance with the circulating strains, universal candidates should induce broad and long-lasting protection against a variety of subtypes. With mRNA, developers can code in conserved internal proteins, including nucleoprotein (NP) and matrix protein (M1), with the potential of cross-protection against both seasonal and pandemic strains [32]. Vaccine activities to combat long-term global health issues have also been revitalized because of the versatility of the platform. Overcoming the barriers to a vaccine in HIV, in which high genetic diversity and immune escape have been in the way of success, candidates of mRNA are under exploration to encode stabilized envelope proteins or sequential immunogens to be used to induce broadly neutralizing antibodies [34]. Clinical trials launched by the National Institutes of Health (NIH) and other consortia indicate the fresh optimism that the speed and modularity of mRNA might at last be used to facilitate effective preventive measures against this tricky virus.

In the case of malaria, the fact that RTS, S/AS01 has only limited effectiveness has prompted the search for multivalent mRNA vaccines to code multiple antigens of *Plasmodium falciparum*. In preclinical work, there is higher antibody breadth and T-cell activation than in the conventional platforms [28]. Plans similar to those of COVID-19 are under development against tuberculosis (TB), where current Bacillus Calmette-Guérin (BCG)-based immunity in adolescents and adults is not strong enough to control the disease. mRNA vaccines that code for *Mycobacterium tuberculosis* proteins are being developed to induce higher Th1 responses that are critical to control the disease [31]. In addition to infectious and neoplastic targets, mRNA technology is being considered in autoimmune and allergic diseases. Immune reprogramming has been shown in mouse models of multiple sclerosis and type 1 diabetes with mRNA constructs that express disease-associated peptides in tolerogenic constructs [27]. The same is being tested in the case of peanut allergy and celiac disease.

Another application is in biodefense and rapid response. The synthetic and cell-free production of the platform allows the quick prototyping of vaccines against emerging zoonoses or potential bioterror agents, e.g., Nipah virus or Marburg virus [45]. Lastly, the mRNA-based interventions are supposed to be further optimized by integration with systems biology, transcriptomics, and AI-aided design. With increased experience of regulatory agencies with nucleic acid vaccines, and when manufacturing capacity is decentralized, the platform will probably transform not only pandemic preparedness but also routine clinical application of immunotherapy [46]. Table 3 summarizes the expanding scope of mRNA vaccine technology, detailing its strategic use across oncology, chronic infections, immune disorders, and biodefense, with an emphasis on mechanisms, development stages, and translational potential.

**Table 3:** Emerging and future applications of mRNA vaccine platforms beyond infectious diseases NSCLC: non-small cell lung cancer; TAAs: tumor-associated antigens; NIH: National Institutes of Health; TB: tuberculosis; BCG: Bacillus Calmette-Guérin; AI: artificial intelligence

Application area	Target/disease	Strategy/mechanism	Development status	Reference
Oncology	Melanoma, NSCLC	Encodes TAAs or patient-specific neoantigens; personalized vaccine design based on tumor sequencing	Clinical trials ongoing	[30]
		Together with immune checkpoint inhibitors (PD-1/CTLA-4), this combination strengthens T-cell-mediated immunity against tumors	Investigational strategy	[10]
Universal influenza	Seasonal and pandemic flu strains	Encodes conserved proteins (e.g., NP, M1) for broad, long-lasting cross-protection across influenza subtypes	Preclinical to early trials	[22]
HIV	HIV-1	Uses stabilized envelope proteins or sequential immunogens to elicit broadly neutralizing antibodies	NIH-supported trials ongoing	[34]
Malaria	Plasmodium falciparum	Encodes multiple antigens to enhance antibody breadth and T-cell activation vs. monovalent RTS, S/AS01	Preclinical studies	[28]
TB	Mycobacterium tuberculosis	mRNA constructs to enhance Th1-type cellular immunity, particularly in adolescents and adults inadequately protected by BCG	Under development	[31]
Autoimmune diseases	Multiple sclerosis, type 1 diabetes	mRNA encodes tolerogenic peptides to induce immune tolerance and reprogramming	Mouse model studies are ongoing	[27]
Allergy	Peanut allergy, celiac disease	Tolerogenic mRNA constructs to modulate hypersensitive immune pathways	Preclinical evaluation	[7]
Biodefense and zoonoses	Nipah virus, Marburg virus	Rapid prototyping of synthetic vaccines for emerging or engineered pathogens	Strategic/preparedness use	[15]
Future directions	Cross-cutting innovation	Integration with systems biology, transcriptomics, AI-aided vaccine design, supported by growing regulatory and decentralized manufacturing frameworks	Ongoing innovation pipeline	[46]

Limitations and future directions in vaccine development

Although the next-generation vaccines, especially the mRNA-based platforms, have great potential, there are a number of limitations that should be addressed. The existing evidence base remains immature, and most of the studies are based on intermediate data and short follow-up, limiting the conclusions about long-term safety and durability. The pandemic has also brought about the possibility of bias in rapid publication since positive results have a higher chance of being published. Also, there is no possibility of comparing trials as they have different study populations, designs, and endpoints. The problems call attention to the demand for long-term follow-up, unified clinical outcomes, as well as consistent worldwide study designs to inform the future formulation of vaccines and policies.

The next steps should be aimed at innovation, equity, and international coordination. Evolution of technologies' advances, like self-amplifying mRNA, thermostable formulations, etc., can allow smaller doses, better scalability, and less reliance on cold-chains, which are key to low-resource contexts. The access can be increased further by improving distribution by means of simplified delivery systems and ambient-stable formats. Harmonization of regulation is necessary to facilitate the process of approvals and emergency authorizations, and it should be safe and transparent. Durable protection against new threats could be provided by investment in broad-spectrum vaccines, such as pan-coronavirus and universal influenza targets. Also, real-time surveillance and digital health infrastructure are critical to monitor the effectiveness of vaccines, vaccine safety, and the evolution of variants.

Policy and implementation implications

The rapid advancement of mRNA and next-generation vaccine technologies underscores the importance of integrated policy frameworks that ensure equitable access, sustainable manufacturing capacity, and harmonized regulatory oversight. Global initiatives must prioritize technology transfer to LMICs, data transparency, and surveillance systems for long-term safety monitoring. Strategic collaboration between governments, academia, and private sectors will be essential to operationalize these innovations for future pandemic preparedness.

## Conclusions

The evolution of vaccine development from classical platforms to mRNA technologies marks a pivotal shift in biomedical innovation. In the face of new infectious threats, the limitations of traditional vaccines, such as live attenuated, inactivated, toxoid, subunit, and viral vector-based, in terms of scalability, production schedules, and cold-chain dependence, have become more apparent. The COVID-19 pandemic sped up the validation of mRNA vaccines, demonstrating their rapidity, adaptability, and promise for preventing infectious diseases and other conditions.

Vaccines must increasingly be treated as global public goods, necessitating frameworks grounded in scientific rigor, ethical distribution, and long-term accessibility. This review uniquely bridges historical, mechanistic, and translational dimensions to present a comparative, cross-platform synthesis of classical and next-generation vaccine modalities. By integrating immunological performance, regulatory pathways, and real-world implementation, it offers a conceptual framework to inform future innovation and global health preparedness. A multidisciplinary strategy linking molecular design, public health ethics, digital surveillance, and policy alignment will be vital to achieving equitable and resilient immunization systems in the post-pandemic era.
